# Supplementary material for: Empirical methods for controlling false positives and estimating confidence in ChIP-Seq peaks
Source: BMC Bioinformatics. 2008 Dec 5;9:523. doi: 10.1186/1471-2105-9-523 (PMC2628906; doi:10.1186/1471-2105-9-523)
Supplement: Additional File 1 — A variety of html documents from the USeq web site detailing the available applications, their best usage, output file type descriptions, command line menus, etc. [file 1471-2105-9-523-S1.zip › USeqUserGuides/JavaDoc.zip!/JavaDoc/util/gen/Email.html]

Email


|  |  |  |
| --- | --- | --- |
| **Overview** \| **Package** \| **Class** \| **Use** \| **Tree** \| **Deprecated** \| **Index** \| **Help** | |  |
| **PREV CLASS**   **NEXT CLASS** | **FRAMES**    **NO FRAMES**     **All Classes** |  |
| SUMMARY: NESTED \| FIELD \| CONSTR \| METHOD | DETAIL: FIELD \| CONSTR \| METHOD |  |


---


## util.gen Class Email

```
java.lang.Object
  util.gen.Email
```

---

``` public class Email extends java.lang.Object ```

---

**Constructor Summary**

`Email(java.lang.String smtpHostName,
java.lang.String smtpUser,
java.lang.String smtpPassword,
java.lang.String MIMEContentType)`


| **Method Summary** | |
| --- | --- |
| `boolean` | `postMail(java.lang.String[] recipients, java.lang.String subject, java.lang.String message, java.lang.String from)` |
| `static boolean` | `postMailNoAuthentication(java.lang.String[] recipients, java.lang.String subject, java.lang.String message, java.lang.String from, java.lang.String MIMEType, java.lang.String SMTPAddress)`             Simple static method for sending email, no authentication, modified from http://www.javacommerce.com/articles/sendingmail.htm Sudhir Ancha. |
| `boolean` | `postWithAttachment(java.lang.String[] recipients, java.lang.String subject, java.lang.String textMessage, java.lang.String fullPathFileName, java.lang.String attachmentFileName, java.lang.String from)` |

**Methods inherited from class java.lang.Object**

`equals, getClass, hashCode, notify, notifyAll, toString, wait, wait, wait`

**Constructor Detail**

### Email

```
public Email(java.lang.String smtpHostName,
             java.lang.String smtpUser,
             java.lang.String smtpPassword,
             java.lang.String MIMEContentType)
```


**Method Detail**

### postMail

```
public boolean postMail(java.lang.String[] recipients,
                        java.lang.String subject,
                        java.lang.String message,
                        java.lang.String from)
```

---


### postWithAttachment

```
public boolean postWithAttachment(java.lang.String[] recipients,
                                  java.lang.String subject,
                                  java.lang.String textMessage,
                                  java.lang.String fullPathFileName,
                                  java.lang.String attachmentFileName,
                                  java.lang.String from)
```

---


### postMailNoAuthentication

```
public static boolean postMailNoAuthentication(java.lang.String[] recipients,
                                               java.lang.String subject,
                                               java.lang.String message,
                                               java.lang.String from,
                                               java.lang.String MIMEType,
                                               java.lang.String SMTPAddress)
```

:   Simple static method for sending email, no authentication, modified from http://www.javacommerce.com/articles/sendingmail.htm Sudhir Ancha.


---


|  |  |  |
| --- | --- | --- |
| **Overview** \| **Package** \| **Class** \| **Use** \| **Tree** \| **Deprecated** \| **Index** \| **Help** | |  |
| **PREV CLASS**   **NEXT CLASS** | **FRAMES**    **NO FRAMES**     **All Classes** |  |
| SUMMARY: NESTED \| FIELD \| CONSTR \| METHOD | DETAIL: FIELD \| CONSTR \| METHOD |  |


---
